# Intracerebral hemorrhage with tentorial herniation: Conventional open surgery or emergency stereotactic craniopuncture aspiration surgery?

**DOI:** 10.1515/tnsci-2020-0173

**Published:** 2021-05-15

**Authors:** Jing Shi, Xiaohua Zou, Ke Jiang, Li Tan, Likun Wang, Siying Ren, Yuanhong Mao, Chunguang Yang, Weijun Wang, Guofeng Wu, Zhouping Tang

**Affiliations:** The Affiliated Hospital of Guizhou Medical University, Postal Address: No. 28, Guiyijie Road, Guiyang City, Postal Code 550004, People’s Republic of China; Department of Neurology, Zhengzhou Second People’s Hospital, Postal address: No. 90, Hanghai Middle Road, Zhengzhou City, Postal Code 450000, Henan Province, People’s Republic of China; Department of Neurosurgery, Qiannan State People’s Hospital of Guizhou Province, Duyun City, Postal Code 558000, People’s Republic of China; Department of Anesthesiology, The Affiliated Hospital of Guizhou Medical University, Postal Address: No. 28, Guiyijie Road, Guiyang City, Postal Code 550004, People’s Republic of China; Emergency Department, The Affiliated Hospital of Guizhou Medical University, Postal Address: No. 28, Guiyijie Road, Guiyang City, Postal Code 550004, People’s Republic of China; Department of Neurology, Tongji Hospital of Tongji Medical College, Huazhong University of Sciences and Technology, Postal address: No.1095, Road Jiefang, Wuhan, Postal code 430030, People’s Republic of China

**Keywords:** hypertensive ICH, tentorial herniation, stereotactic minimally invasive surgery, conventional craniotomy, secondary epilepsy

## Abstract

**Background:**

To observe the therapeutic effect of conventional decompressive craniectomy with hematoma evacuation and frame-based stereotactic minimally invasive surgery (MIS) for supratentorial intracranial hematoma with herniation.

**Methods:**

One hundred forty-nine patients with hypertensive ICH complicated with tentorial herniation were reviewed and analyzed in the present study. The intracranial hematoma was evacuated by emergency surgery within 6 h after admission. According to the authorized representatives’ wishes and consent, 74 of the 149 patients were treated by conventional decompressive craniectomy followed by hematoma removal, defined as the CDC group, and the remaining 75 patients underwent frame-based stereotactic MIS for ICH evacuation, defined as the MIS group. The intervals between the admission to surgery, the duration of surgery, the amount of iatrogenic bleeding, the occurrence of postoperative rebleeding, and the recovery of neurological functions were compared between the two groups. All patients were followed up for 3 months. Secondary epilepsy, survival in a vegetative state, severe pulmonary complications, mortality, and activities of daily living (ADL) classification were also recorded and compared.

**Results:**

The interval between admission and surgery, the duration of surgery, and intraoperative blood loss in the MIS group were significantly decreased compared to the CDC group. The mortality rate, the rate of rebleeding, prevalence of vegetative state, and severe pulmonary complications in the MIS group were remarkably decreased compared to the CDC group. In the MIS group, the survivors’ (ADL) grade also showed advantages.

**Conclusions:**

In the surgical treatment of hypertensive ICH complicated with tentorial herniation, frame-based stereotactic MIS for ICH showed advantages compared to conventional open surgery.

## 1 Background

Hypertensive intracerebral hemorrhage (ICH) remains the most devastating type of stroke and is a leading cause of disability and mortality. Few evidence-based targeted treatments exist for ICH, in contrast with advances in ischemic stroke treatment [1]. A possible advantage of surgical treatment over conservative treatment of spontaneous hypertensive ICH is considered controversial [2]. Patients with spontaneous supratentorial ICH in neurosurgical units showed no overall benefit from early surgery when compared with initial conservative treatment [3]. An international, parallel-group trial undertaken in 78 centers in 27 countries showed that early surgical procedures might have a small but clinically relevant survival advantage for patients with spontaneous superficial ICH without intraventricular hemorrhage [4]. However, large supratentorial ICH is usually complicated by tentorial herniation, and emergency surgery for ICH evacuation is urgent for patients with ICH complicated by cerebral herniation. Several types of surgical techniques, such as stereotactic minimally invasive surgery (MIS) for hematoma aspiration, conventional decompressive craniectomy followed by ICH evacuation, and small bone window craniotomy with cranial catheterization, are available for patients with large volume ICH [5]. However, subcortical injury resulting from conventional surgical management of ICH may counteract the potential benefits of hematoma evacuation during open surgery [6]. The traditional view is that a small bone window craniotomy cannot effectively reduce intracranial pressure, relieve cerebral hernia, improve the circulation of brain blood, and effectively stop bleeding because it can only be used to remove a small portion of a hematoma and may lead to rebleeding. Therefore, hematoma aspiration was not considered to be an effective treatment for hypertensive ICH complicated with brain herniation [6]. In recent years, minimally invasive techniques for ICH evacuation have been developed in neurosurgery and have achieved promising results in patients with hypertensive ICH complicated with cerebral herniation [7]. A recent retrospective study demonstrated that MIS was safe and effective in patients with hypertensive ICH with a hematoma volume >50 mL and was considered to be superior to craniotomy [8]. Hanley and his colleagues performed a clinical trial on safety and efficacy of MIS plus alteplase in ICH evacuation; their results could lead to the addition of surgical management as a therapeutic strategy for ICH [9]. The hematoma size reduction to 15 mL or less was associated with improved mRS scores at 365 days [10]. Hemispheric large volume hematoma complicated by tentorial herniation is a critical and life-threatening condition. Surgery might be the only option to save patients’ lives. However, the efficacy of emergent MIS for patients with large hematomas complicated with tentorial herniation is unclear. The present study aimed to observe the therapeutic effects of emergent MIS for patients with hemispheric large volume ICH complicated with tentorial herniation. Patients who received conventional decompressive craniectomy for ICH clearance were used as the controls.

## 2 Methods

### 2.1 Patients

Patients with ICH complicated with tentorial herniation who were admitted to the Affiliated Hospital of Guizhou Medical University, Qiannan State People’s Hospital, and Zhengzhou Second People’s Hospital of Henan Province between January 2014 and October 2017 were included in our study.

The inclusion criteria for the present study were as follows: (1) patients (ages greater than 18 years) who had a history of hypertension or hypertension observed upon admission, and the symptoms and signs met the diagnostic criteria for ICH, which was confirmed by a nonenhanced CT scan; (2) patients who suffered from supratentorial spontaneous ICH, including ICH in the basal ganglia, thalamus, subcortex, or cerebral lobe; (3) patients who were complicated with tentorial herniation; and (4) patients with no contraindications for surgery. Authorized representatives of the patients were consented to approve the surgery.

The exclusion criteria were as follows: patients with ICH due to head trauma, brain tumor, aneurysm, or malformation, those receiving anticoagulant therapy and/or antiplatelet drugs, or those who had secondary ICH from hemorrhagic transformation of a brain infarction were excluded from the study. Patients with ICH located in the brainstem and cerebellum and those patients who were not suitable for surgery or for whom their authorized representative did not consent to surgery were also excluded from our study.

The patients were diagnosed by baseline CT scan within 1 h, and the surgery was performed within 6 h after admission. The follow-up CT scan was performed at least twice in the following three days after surgery. Demographic information and time to baseline and follow-up CT scans were recorded for each patient.

Based on the inclusion criteria, a total of 149 sequential patients with supratentorial spontaneous ICH complicated with tentorial herniation were included in the present study. The neurosurgeons informed the authorized representatives of the advantages and disadvantages of each surgical procedure. The decision about which surgical strategy to use was made by the authorized representatives, and informed consent was obtained. According to the authorized representatives’ wishes, 74 patients were assigned to the conventional decompressive craniectomy followed by ICH evacuation group (CDC group, *n* = 74), and 75 patients were allocated to the frame-based stereotactic MIS for ICH evacuation group (MIS group, *n* = 75). The basic data are listed in Table 1. No statistically significant differences in terms of age, sex, hypertension, hematoma volume, hematoma location, neurological functions, etc. were observed between the groups.

**Table 1:** Comparison of basic data between patients receiving MIS and patients after CDC treatment

Characteristics	CDC group (*n* = 74)	MIS group (*n* = 75)	*P* value	*t*/*χ* ^2^
Mean age (year, \bar{X}] ± s)	63.4 ± 12.5	61.5 ± 10.7	0.320	0.996
Sex, male (*n*, %)	45 (60.8)	43 (57.3)	0.666	0.186
Smoking (*n*, %)	32 (43.2)	34 (45.3)	0.797	0.066
Hypertension (*n*, %)	51 (68.9)	53 (70.7)	0.816	0.054
Diabetes mellitus (*n*, %)	11 (14.9)	10 (13.3)	0.788	0.072
Alcohol consumption (*n*, %)	15 (20.2)	16 (21.2)	0.873	0.026
Systolic pressure (\bar{X}] ± s)	178.2 ± 26.3	175.8 ± 27.1	0.584	0.549
Diastolic pressure (mm Hg, \bar{X}] ± s)	106.3 ± 18.9	108.5 ± 19.6	0.489	0.698
Time to baseline CT (h, \bar{X}] ± s)	8.3 ± 4.3	8.1 ± 4.2	0.774	0.287
Baseline ICH volume (mL, \bar{X}] ± s)	74.3 ± 24.8	69.6 ± 20.2	0.207	1.267
Glasgow coma scale s (points, \bar{X}] ± s)	4.6 ± 2.3	4.5 ± 2.7	0.808	0.244
NIHSS on admission (points, \bar{X}] ± s)	32.0 ± 4.5	31.7 ± 4.6	0.688	0.402
**ICH in the right hemisphere (** ***n*** **, %)**	32 (43.2)	35 (46.7)	0.675	0.176
Ganglia area (*n*, %)	25 (78.1)	29 (71.4)	0.625	0.239
Thalamus (*n*, %)	3 (9.4)	1 (2.8)	0.342	0.370
Cerebral lobes (*n*, %)	4 (12.5)	5 (14.3)	1.000	0.000
**ICH in the left hemisphere (** ***n*** **, %)**	42 (56.8)	40 (53.3)	0.675	0.176
Ganglia area (*n*, %)	31 (73.8)	33 (82.5)	0.342	0.903
Thalamus (*n*, %)	4 (9.5)	2 (5)	0.676	0.131
Cerebral lobes (*n*, %)	7 (16.7)	5 (12.5)	0.594	0.285

CT indicates computed tomography; ICH indicates intracerebral hemorrhage; NIHSS indicates National Institutes of Health Stroke Scale; CDC indicates conventional decompressive craniectomy; MIS indicates minimally invasive surgery.


**Ethical approval:** The present study was approved by the ethics committee of the Affiliated Hospital of Guizhou Medical University and was performed in compliance with the WMA Declaration of Helsinki–Ethical Principles for Medical Research Involving Human Subjects.
**Informed consent:** All the patients’ authorized representatives and those patients who had the ability to communicate with the doctors agreed to participate in the study. The informed consent was obtained in written form.

### 2.2 Conventional open surgery for ICH clearance

**Figure 1:**
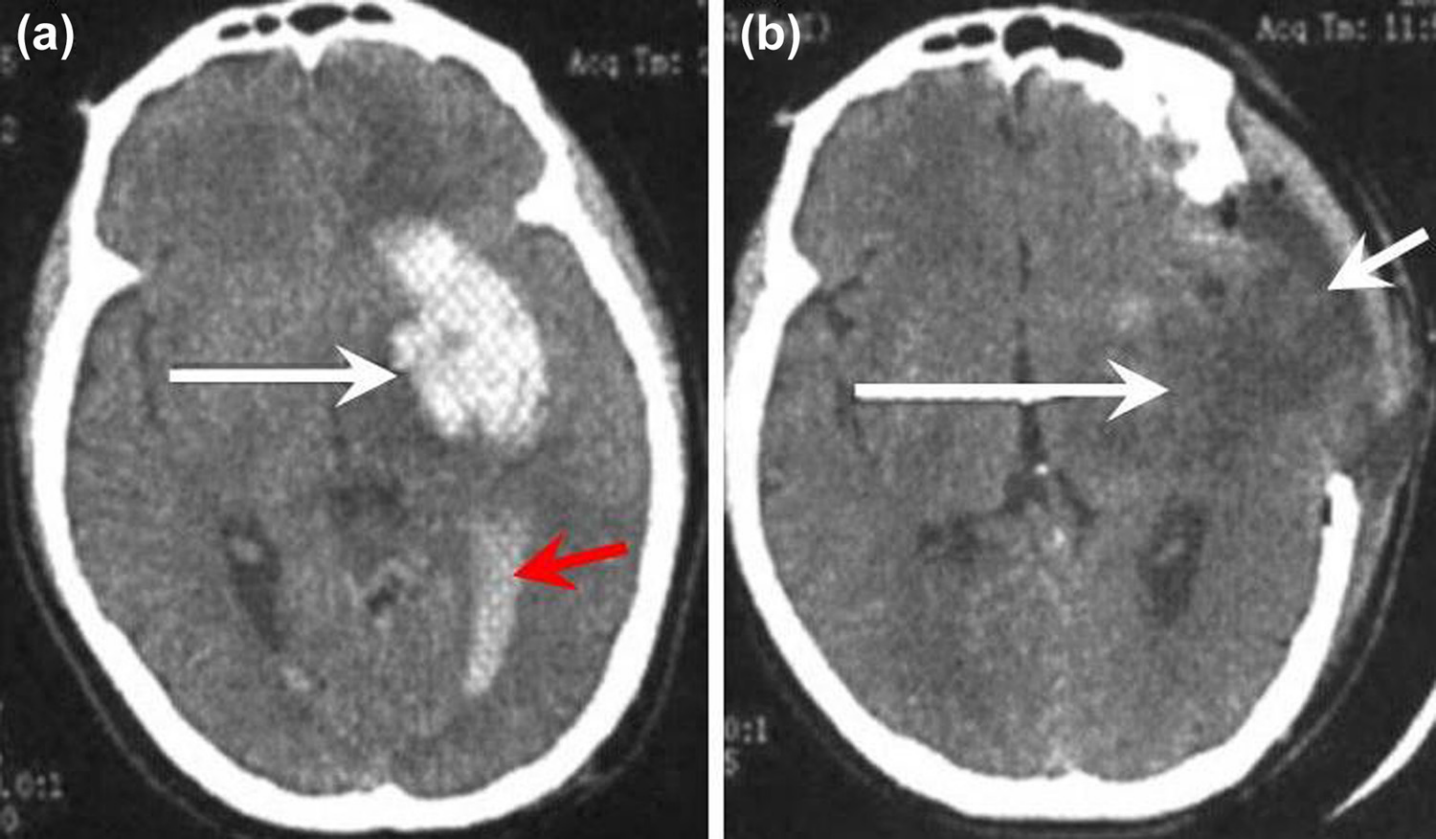
Hematoma changes after conventional decompressive craniectomy. (a) The white arrowhead pointed to the large supratentorial hematoma with a volume more than 50 mL. The red arrowhead indicated the intraventricular hemorrhage. The brain tissues around the ICH were oppressed. The patient was comatose and the diameter of the pupil on the hematoma side dilated to 4 mm (the contralateral pupil diameter was 2.5 mm). (b) The hematoma was removed by conventional open surgery (the long arrowhead), the brain edema was remarkable, and the edematous brain protruded out of the skull through a skull defect (the short arrowhead).

After the patient received general anesthesia, the neurosurgeons removed the bone flap and opened the dura according to the hematoma location. The hematomas were evacuated as much as possible. A drainage tube was placed in the residual hematoma or subdural region according to the hematoma volume and intraoperative condition. The patients with hemorrhage that had ruptured into the ventricles underwent external ventricular drainage prior to craniotomy. After confirmation of no bleeding in the hematoma area, an intracranial pressure probe was placed in the ventricles, the skin was sutured, and the drainage tube was fixed. The patients were transferred to the neurosurgery intensive care unit (NICU) after surgery. The drainage tube was removed based on the volume of drainage within 24–72 h. A follow-up CT scan was performed three days after surgery (Figure 1).

### 2.3 Frame-based stereotactic MIS for ICH evacuation

The MIS for the ICH evacuation was similar to the procedure that was used in our previously published studies [11,12]. Briefly, each patient underwent a CT scan after a stereotactic positioning headframe was fixed on the patient’s head. The coordinates of the targets were determined using CT data on the computer. After obtaining the coordinates of the targets, an arc frame and guider were fixed on the headframe, and the parameters were set. After local anesthesia and intravenous sedation and under the guidance of the arc frame and the guider, a YL-1 craniopuncture needle (inner diameter 2.5 mm, external diameter 3 mm) (Beijing WanTeFu Medical Apparatus Co., Ltd, Beijing, China) was driven into the skull using an electric drill inserted to the expected depth, which was predesigned to ensure that the skull was penetrated while not injuring the brain parenchyma. Then, plastic blunt stylets, 2 mm longer than the needles, were inserted to replace the metal needle stylets to make the endpoint blunt. Gently and carefully, the puncture needles were then advanced to the targets manually. Removing the plastic needle core, the liquid part of the hematoma was aspirated with a 10 mL syringe that was connected to the LY-1-type needle system. The hematoma area was flushed with sterile saline 2 to 3 times using a high-pressure jet-washing device. Subsequently, the drainage bags were connected to the needle for ICH drainage following administration of urokinase. The urokinase (50,000 units diluted in 2 mL of normal saline) was injected slowly every 8 h into the residual hematoma area to dissolve the solid part of the hematoma. The needle system was closed for 2 h before reopening to allow spontaneous drainage. After the operation, the patient received an immediate postoperative CT scan to inspect the location of the needles.

**Figure 2:**
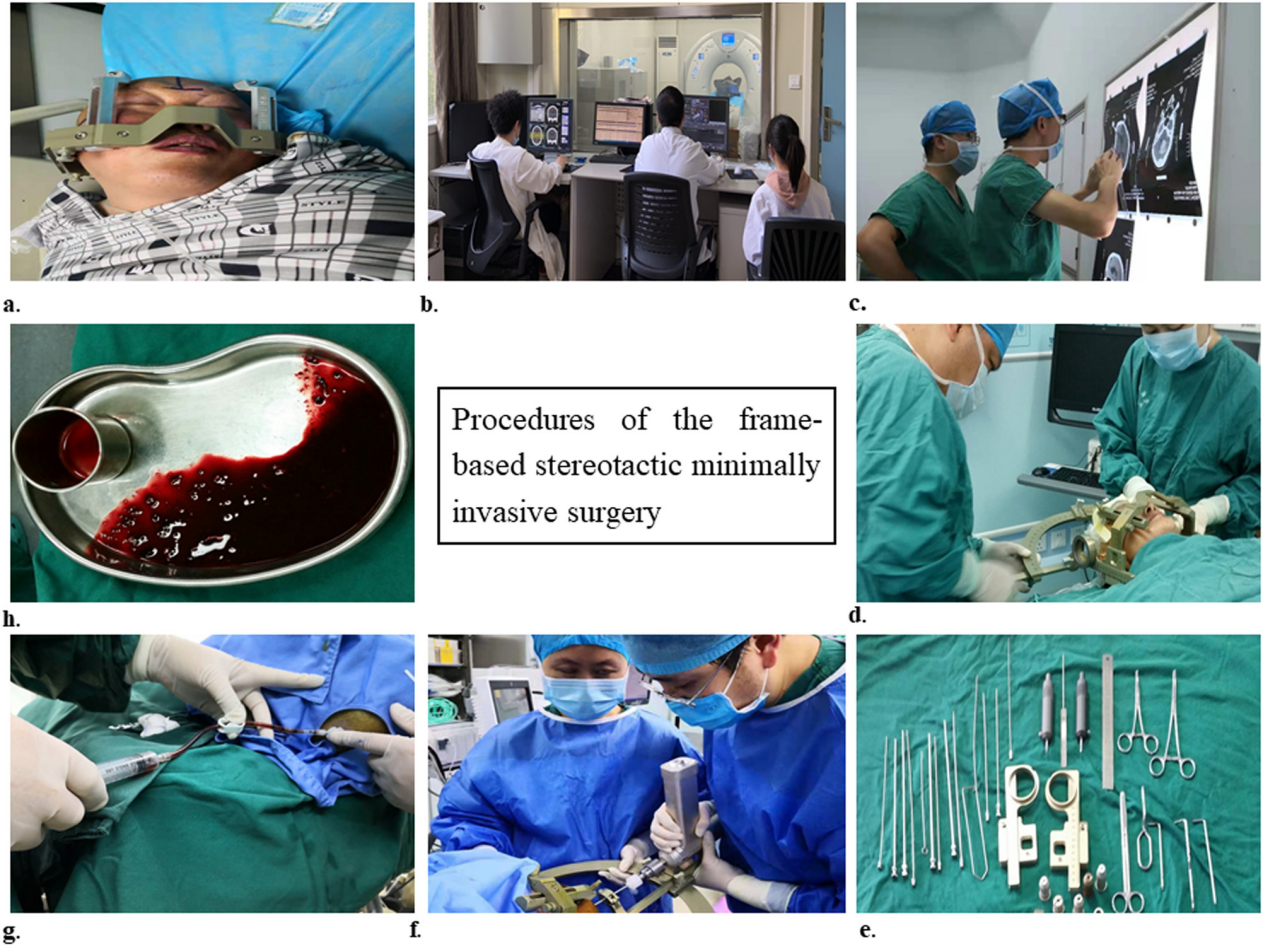
Procedures for the stereotactic minimally invasive surgery. A positioning headframe was fixed on the head first and then the patient was transferred to scan CT for figuring out the coordinates (a–c). Subsequently, the arc frame and guider were fixed to the positioning headframe and a transcranial puncture needle was inserted (d–f). Finally, the liquid part of the ICH was aspirated out (g and h). The puncture needle set and the drainage system were composed of a needle guard, a metal needle core, a plastic needle core, and a plastic drainage tube.

The patients were transferred to the intensive care unit after removing the location framework and stereotactic apparatus. The LY-1-type puncture needle system was removed after the hematoma was either completely or nearly completely evacuated (Figure 2). Generally, the postoperative follow-up CT scan was performed on the first day (the first postoperative CT) and the third day (the second postoperative CT) after surgery. If the patients showed deterioration of the symptoms at any time after surgery, repeat CT was performed.

### 2.4 Medications

All patients in our study received the same medical management based on the guidelines for the treatment of hypertensive ICH, including management of coagulopathy and blood pressure and prevention and control of secondary brain injury and intracranial pressure [13]. In addition, more comprehensive measures were also taken, including prevention of deep venous thrombosis, the control of temperature and blood glucose, nutritional support, and the prevention of other complications. In the control group patients, hypertension was properly controlled, and all patients were followed up for 3 months.

### 2.5 Efficacy evaluation

#### 2.5.1 Evaluation indices

The time intervals between admission and surgery, duration of surgery, volume of iatrogenic blood loss during operation, rate of postoperative intracerebral rebleeding, rate of secondary epilepsy, survival rate in a vegetative state, serious pulmonary complications, neurological functions evaluated by the National Institutes of Health Stroke Scale (NIHSS), Glasgow Coma Scale score (GCS), mortality rate, and activities of daily living scale scores (ADL) were recorded and analyzed. ADL score grades were classified as I–IV. The number of cases and percentage of patients within each grade were recorded. The amount of iatrogenic bleeding was calculated based on the dry-wet gauze weight, the volume in the suction apparatus, and the flushing fluid.

### 2.6 Judgement of postoperative rebleeding

**Figure 3:**
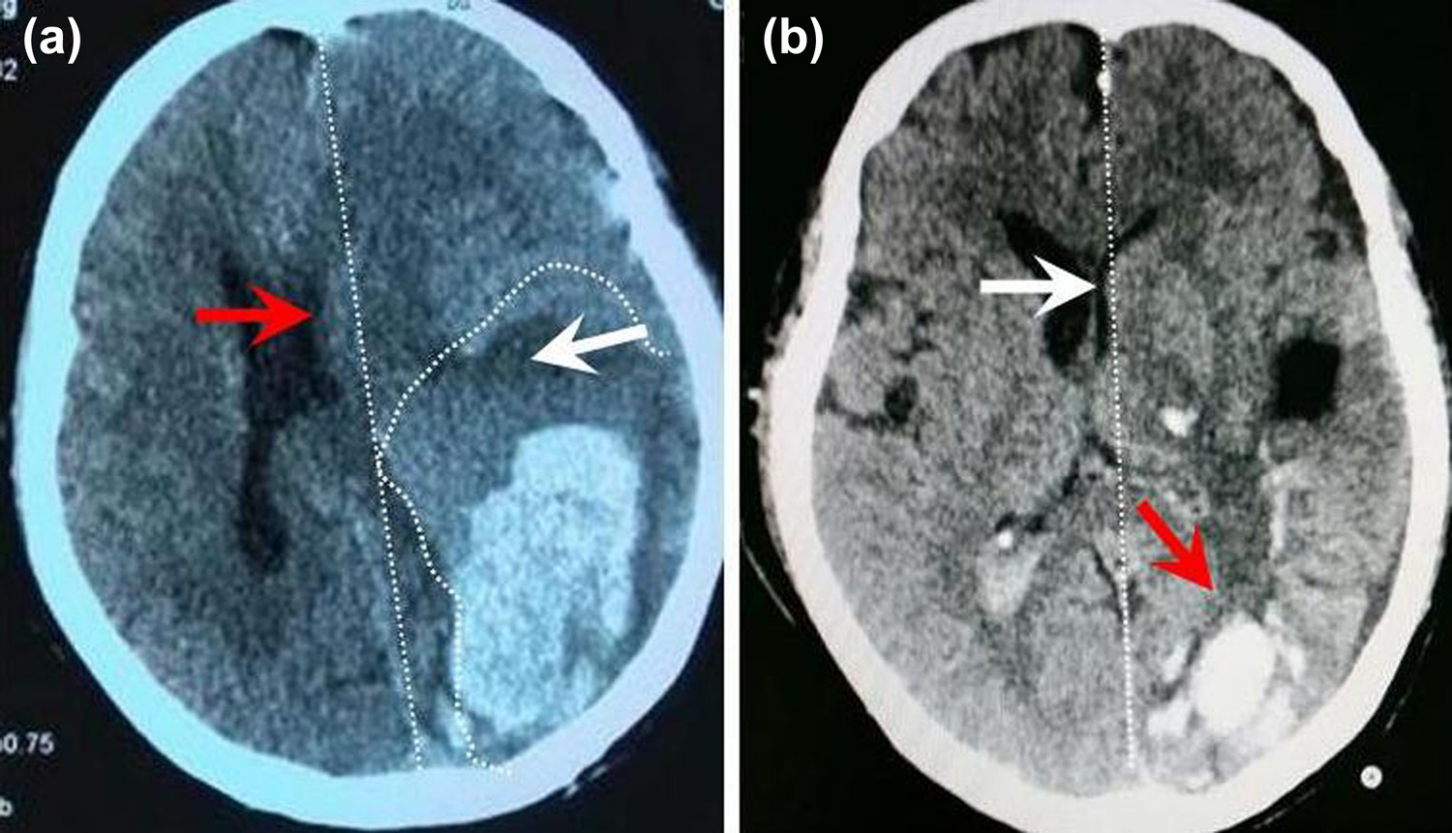
Hematoma changes after stereotactic minimally invasive surgery. (a) Indicates a large supratentorial hematoma with a volume more than 100 mL. The brain tissues were oppressed by the ICH and the perihematomal edema was severe (the white arrowhead). The midline structures shifted to the contralateral side (the red arrowhead). The patient was comatose on admission and the diameter of the pupil on the hematoma side dilated to 4.5 mm (the contralateral pupil diameter was 3 mm). (b) Indicates that most of the hematoma was evacuated by an emergent stereotactic minimally invasive surgery (the red arrowhead). The midline shift decreased (the white arrow). The dilated pupil recovered to normal level 1 h after surgery and the patient recovered to consciousness on the following day.

The following situations suggested postoperative intracerebral rebleeding: (A) exacerbation of disturbance of consciousness; (B) the pupil on the ICH side dilated again after recovering to normal size; (C) monitors showed that ICP increased suddenly and significantly; and (D) based on the criteria used in several large clinical studies on ICH, we defined intracranial postoperative rehemorrhage as an increase in the hematoma volume of >33% [14] compared to the last previous CT scan (on which the ICH volume had decreased significantly after MIS) or the reappearance of hyperdensity in the focal region at the follow-up CT scan (Figure 3) after it was removed completely following surgery [15]. Hematoma volumes were estimated based on CT using the ABC/2 formula (*t* = *π*/6 × *l* × *s* × slice) [16].

### 2.7 Definition of secondary epilepsy

Secondary epilepsy was defined as Generalized seizures that occurred more than twice during the follow-up period. Seizures were classified according to their time of onset. Seizures occurring within 1 week after stroke were classified as early seizures, and seizures occurring thereafter were classified as late seizures [17].

### 2.8 Pulmonary complications

Some patients suffered from life-threatening complications during their hospital stay. Severe complications included severe pulmonary infection and respiratory failure.

### 2.9 Activity of daily living scale (ADL) [18]

Patients’ recovery was estimated based on outcomes at follow-up within 3 months after surgery. Grade I shows that the patients have complete recovery of muscle strength and ADL ability. Grade II manifests recovery of segmental daily living ability, living independently. Grade III indicates that the patients need maximal assistance to live in the community, but could walk with a crutch. Grade IV indicates that the patients are bedridden with consciousness. Grade V indicates the vegetative state. Grades I–III indicate the best prediction of independence in the community. Grades IV–V indicate poor ability in ADL.

### 2.10 Statistical method

Statistical analysis was performed with SPSS 21.0 statistical software. Categorical data are shown as proportions, and continuous variables are presented as the mean ± SD. Comparisons between two sample means were conducted with the *t*-test after homogeneity tests of variances. Chi-square tests and Fisher’s exact tests were used to compare the rates between groups. One-way analysis of variance was performed to compare across groups. An STD method was performed to detect a difference between any two groups when a total difference was observed among groups. A *P* < 0.05 indicates that the difference was statistically significant.

## 3 Results

### 3.1 Time of surgery and the amount of iatrogenic blood loss

**Figure 4:**
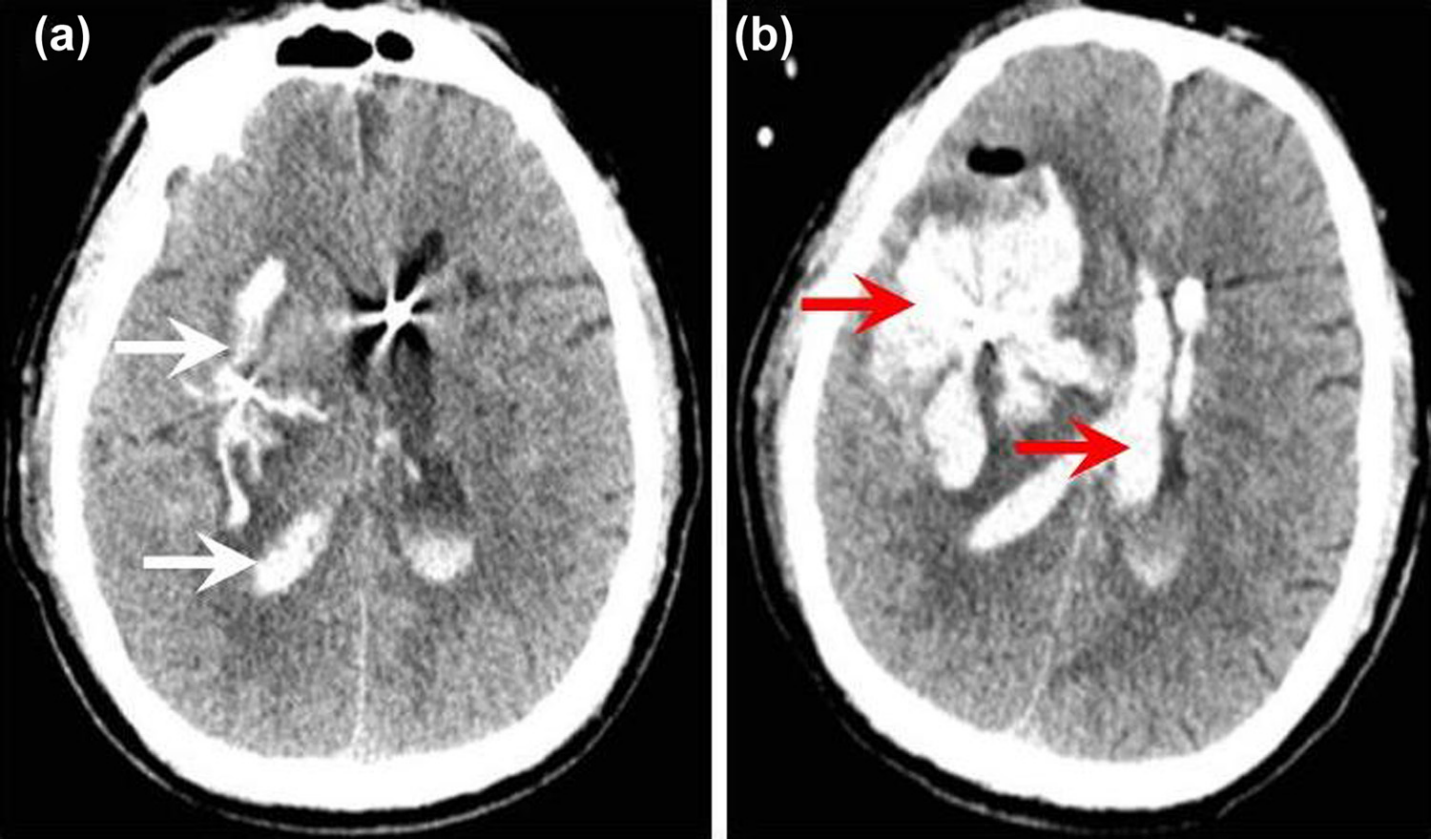
Postoperative intracranial rebleeding in patients underwent surgery. (a) A small amount of residual hematoma in the brain and the ventricles were observed on postoperative CT follow-up (the white arrowhead). (b) The intracerebral hemorrhage and the intraventricular hemorrhage increased significantly (the red arrowhead) compared with the previous follow-up CT on which the residual hematoma volume was very small (a). A significant increase in midline shift has also been noted.

**Figure 5:**
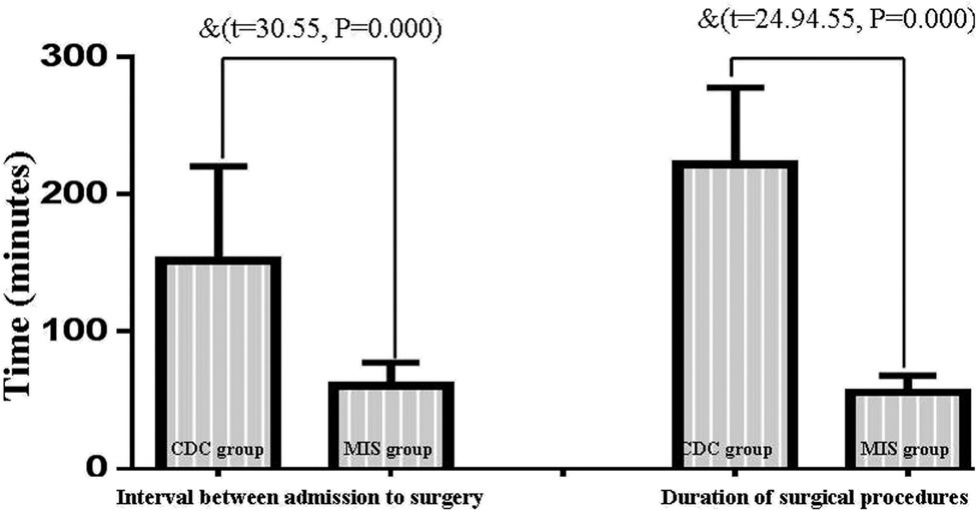
The comparison of the time indices between the MIS group and the CDC group. The time interval between the admission and the beginning of surgery decreased significantly in the MIS group compared to the CDC group (^&^decreased as compared with CDC group, *P* < 0.05). The duration of surgery was also shortened in the MIS group (^&^decreased as compared with CDC group, *P* < 0.05).

The time interval between admission and surgery and the duration of surgery in the MIS group were significantly decreased compared with the CDC group (Figure 4). The amount of intraoperative bleeding in the CDC group was significantly increased compared to the MIS group (Figure 5).

### 3.2 Short-term outcomes

We compared the NIHSS and the GCS between the two groups at each time point. The postoperative neurological functions were improved remarkably in each group compared with those on admission. However, at each time point within three months, the neurological functions of survivors recovered better in the MIS group compared with the CDC group (Table 2, *P* < 0.05). The GCS scores at one and two weeks after surgery also improved significantly in the two groups. (Table 3, *P* < 0.05). However, the GCS increased significantly at one and two weeks after surgery in the MIS group, but not at one month and thereafter compared with the CDC group. These results suggested that the CDC and the MIS could improve the GCS and that the MIS could promote the recovery of consciousness in a short time. The final mortality rate and the survival rate in a vegetative state in the MIS group were significantly decreased compared with the CDC group. In addition, activity of daily living ability in the MIS group was also significantly more advantageous than in the CDC group. The number of patients with ADL grades I–III increased, and patients with ADL grade IV decreased in the MIS group compared with those who underwent conventional open surgery (Table 4, *P* < 0.05).

**Table 2:** Comparison of neurological functions (NIHSS) after surgery (mean ± SD)

Group	Admission	1 week	2 weeks	4 weeks	12 weeks	*F*	*P*
	*n* = 74/75	*n* = 67/69	*n* = 54/67	*n* = 45/66	*n* = 38/64		
CDC group	32.0 ± 4.5	23.7 ± 3.0^$^	18.9 ± 2.6^$**ʘ**^	16.4 ± 2.5^$**ʘ#**^	13.6 ± 2.2^$**ʘ#**&^	287.6	0.000
MIS group	31.7 ± 4.6	17.9 ± 2.3*^$^	14.3 ± 2.0*^$**ʘ**^	11.3 ± 2.1*^$**ʘ #**^	8.7 ± 2.5*^$ʘ#&^	681.6	0.000
*t*	0.402	12.680	11.000	11.621	9.997		
*P*	0.688	0.000	0.000	0.000	0.000		

*Decreased compared with the CDC group, *P <* 0.05. ^$^Decreased compared with the admission, *P <* 0.05. ^ʘ^Decreased compared with 1 week after surgery, *P <* 0.05. ^#^Decreased compared with 2 weeks after surgery, *P <* 0.05. ^&^Decreased compared with 4 weeks after surgery, *P <* 0.05. CDC indicates conventional decompressive craniectomy; MIS indicates minimally invasive surgery; *n* = CDC/MIS.

**Table 3:** Changes of Glasgow Coma Scale scores (mean ± SD)

Group	Admission	1 week	2 weeks	4 weeks	12 weeks	*F*	*P*
	*n* = 74/75	*n* = 67/69	*n* = 54/67	*n* = 45/66	*n* = 38/64		
CDC group	4.6 ± 2.3	6.9 ± 2.3^$^	8.6 ± 2.5^$**ʘ**^	10.4 ± 2.8^$**ʘ#**^	12.0 ± 2.4^$**ʘ#**&^	76.43	0.000
MIS group	4.5 ± 2.7	8.8 ± 2.4*^$^	10.6 ± 2.3*^$**ʘ**^	12.1 ± 3.1*^$**ʘ#**^	13.8 ± 2.6*^$**ʘ#**&^	129.4	0.000
*t*	0.243	4.711	4.574	2.948	3.477		
*P*	0.808	0.000	0.000	0.004	0.001		

*Decreased compared with the CDC group, *P <* 0.05. ^$^Decreased compared with admission, *P <* 0.05. ^ʘ^Decreased compared with 1 week after surgery, *P <* 0.05. ^#^Decreased compared with 2 weeks after surgery, *P <* 0.05. ^&^Decreased compared with 4 weeks after surgery, *P <* 0.05. CDC indicates conventional decompressive craniectomy; MIS indicates minimally invasive surgery; *n* = CDC/MIS.

**Table 4:** Comparison of survivor status between two groups (*n*, %)

Group	*n*	Vegetative state	ADL-I	ADL-II	ADL-III	ADL-IV
CDC group	74	13 (17.6)	4 (5.5)	3 (4.0)	7 (9.5)	11 (14.9)
MIS group	75	3 (4.0)*	24 (32.0)^&^	19 (25.3)^&^	16 (21.3)^&^	2 (2.8)*
*χ* ^2^		7.153	17.263	13.402	4.023	6.960
*P*		0.007	0.000	0.001	0.045	0.008

*Decreased compared with the CDC group, *P <* 0.05. ^&^Increased compared with the CDC group. CDC indicates conventional decompressive craniectomy; MIS indicates minimally invasive surgery; ADL indicates activity of daily living.

### 3.3 Main complications

**Figure 6:**
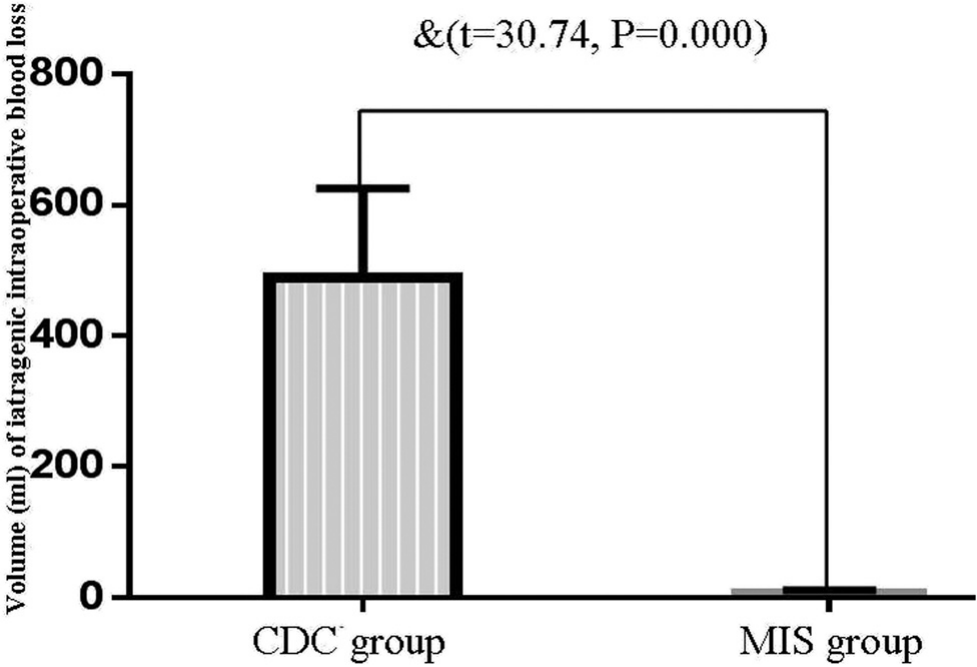
The comparison of the iatrogenic intraoperative blood loss between the MIS group and the CDC group. Only a few amount of iatrogenic intraoperative blood loss was noted in the MIS group. The amount of iatrogenic intraoperative blood loss in the CDC group increased significantly compared to the MIS group (^&^increased as compared with MIS group, *P* < 0.05).

During the hospital stay, 11 patients had complicated postoperative rebleeding (only three patients received a second surgery) and 24 patients suffered severe pneumonia. Seventeen patients were manifested with generalized seizures during three months of follow-up. Compared with the CDC group, the incidence of rebleeding and secondary epilepsy was decreased in the MIS group (Table 5, *P* < 0.05), suggesting that the MIS could reduce the incidence of severe complications compared to the CDC. No significant difference was observed in severe pulmonary complications between the two groups (Figure 6).

**Table 5:** Incidence of severe complications and mortality between two groups (*n* (%))

Group	Postoperative rebleeding	Secondary epilepsy	Pulmonary infection and respiratory failure	Mortality
CDC group	9/74 (12.1)	13/74 (17.6)	16/74 (21.6)	36/74 (48.6)
MIS group	2/75 (2.7)*	4/75 (5.3)*	8/75 (10.7)	11/75 (14.7)*
*χ* ^2^	4.912	5.516	3.308	19.92
*P*	0.027	0.019	0.069	0.000

*Decreased as compared with the CDC group, *P <* 0.05. CDC indicates conventional decompressive craniectomy; MIS indicates minimally invasive surgery compared with MIS group, *P* < 0.05.

## 4 Discussion

Spontaneous hypertensive ICH, the most devastating and debilitating form of stroke, remains a major healthcare concern all over the world [19]. In addition to a higher mortality, these strokes are often associated with severe neurological impairment for those affected [20]. An estimated mortality rate for ICH reached approximately 40% within one month following the onset of the disease, and only 12–39% of the survivors could perform ADL at the time of hospital discharge [21]. In patients with large volume ICH complicated by cerebral herniation, the fatality and disability rates are very high. How to reduce the mortality and disability of ICH patients with cerebral herniation remains a great challenge in clinical practice. However, emergent medications did not show an ideal effect for such patients [22].

Surgical procedures are necessary to save the life of patients with large ICH complicated by cerebral herniation. The ultra-early surgeries performed before perihematomal ischemia, necrosis, and edema occur, removing most of the hematoma and reducing the compression of hematoma, are believed to be the optimal way to minimize the damage to brain tissue and prevent the deterioration of brain edema [23]. In the present study, conventional open surgery and stereotactic craniopuncture aspiration followed by thrombolysis were used in the treatment of patients with large volume ICH complicated with tentorial herniation. The results demonstrated that mortality was significantly reduced and quality of life was improved in patients who received the minimally invasive procedures for ICH removal. The incidence of complications, such as postoperative rebleeding, secondary epilepsy, and severe pulmonary complications, decreased remarkably. Stereotactic craniopuncture aspiration could also significantly shorten the interval between admission and surgery and the duration of surgery and reduce iatrogenic blood loss in our study. It could quickly puncture the hematoma and aspirate out the liquid parts of the ICH, reducing the intracranial pressure in a very short time. The residual half solid hematoma could be resolved by fibrinolytic agents. The resolved hematoma could be finally evacuated.

Postoperative rebleeding remains a great challenge in patients undergoing surgery. In the present study, the incidence of postoperative rehemorrhage was 6.7% in the MIS group and 10% in the CDC group, suggesting that the MIS could decrease postoperative intracerebral rebleeding. These results were similar to previously published studies, in which the incidence of rehemorrhage was 10.0% in patients who underwent MIS and 15.4% in those who underwent conventional craniotomy [24]. Frame-based stereotactic aspiration and subsequent fibrinolytic therapy using urokinase for spontaneous ICH is a simple and safe procedure with low mortality and rebleeding rate [25].

Seizures are a common complication after an ICH, and epilepsy might even be drug-resistant [26]. Poststroke epilepsy could severely affect the patients’ quality of life. In a newly published study, 13.5% of patients with ICH developed poststroke epilepsy. The risk factors for poststroke epilepsy included subcortical hematoma location, larger hematoma volume, and hematoma evacuation [27]. In the present study, all three risk factors existed. The total incidence of poststroke epilepsy was 14.5%, which was similar to the abovementioned reports. However, the incidence of poststroke epilepsy was only 3.3% in patients who underwent MIS, but 22.0% in those patients who received conventional open surgery. These results suggested that the MIS could also decrease the incidence of poststroke epilepsy because the minimally invasive procedures minimized the iatrogenic injuries to the normal brain overlying the ICH.

Severe pulmonary complications, such as severe pneumonia-induced respiratory failure, are important factors threatening the patients’ life after removing the threat of brain hernia by surgery for large volume ICH evacuation. In previously published studies, 7/62 patients (11.2%) died of respiratory failure and one patient (1.6%) died of heart disease [27]. In our study, the incidence of severe pulmonary complications was 3/33 (9.1%) in MIS-treated populations, but 9/50 (18.0%) in those who received conventional craniotomy for hematoma removal. Compared with conventional open surgery, the MIS decreased the incidence of severe pulmonary complications. Stereotactic aspiration of ICH improves the general condition of the patients, promotes improvement of consciousness, and decreases the incidence of pneumonia [28]

Conventional craniotomy for hematoma removal plus bone flap decompression is commonly used for patients with large volume hematoma. Previous studies demonstrated that it could reduce the intracranial pressure, prevent and reduce blood cell decomposition after bleeding, increase the survival rate, and improve the quality of life [29,30]. However, iatrogenic injuries of conventional open surgery remain serious. Mortality and the rate of long-term complications, such as secondary epilepsy, remain very high. The long course of surgery, serious trauma, high anesthesia risk, and many postoperative complications demonstrated that the therapeutic effects are not ideal [31].

Stereotactic craniopuncture aspiration followed by thrombolysis has emerged as a promising strategy for modifying the neurological outcome of patients with ICH [8,31]. However, the efficacy of such procedures in patients with large volume ICH complicated with tentorial herniation is poorly reported. Our results demonstrated that the MIS might be safe and effective for large volume supratentorial ICH patients with tentorial herniation. A recent retrospective study also obtained similar results, in which the authors demonstrated that the MIS was safe and effective in patients with an intracerebral hematoma volume >50 mL and was considered to be superior to craniotomy [8].

In conclusion, for patients with hypertensive hemispheric ICH complicated with tentorial herniation, emergency stereotactic craniopuncture aspiration followed by thrombolysis could decrease mortality and the rate of vegetative state, reduce the rate of severe complications, and improve the quality of life of survivors.

## Abbreviations

ICH: intracerebral hemorrhage
CT: computed tomography
sMIS: stereotactic minimally invasive surgery
GCS: glasgow coma scale
NIHSS: National Institute of Health Stroke Scale
CDC: conventional decompressive craniectomy
ADL: activity of daily life.
